# Microgranular Skin Grafting for a Sacral Pressure Injury: A Case Report

**DOI:** 10.7759/cureus.87969

**Published:** 2025-07-15

**Authors:** Baiying He, Min Yang, Bin Wen

**Affiliations:** 1 Department of Surgery, Nan Jiao Hospital of Chongqing City (Daojiao Community Healthcare Center), Chongqing, CHN

**Keywords:** case report, microgranular skin grafting, postoperative care, pressure injury, spinal injury

## Abstract

Pressure injury (PI) is an adverse event and a global issue. This case report examines the efficacy of microgranular skin grafting in treating a sacral stage III pressure injury in a 49-year-old male patient with paraplegia due to a severe car accident 15 years prior. Conventional wound management had failed, leading to worsening ulcers. After thorough debridement and preparation, microgranular skin grafting was performed, resulting in complete epithelialization within 30 days. The patient's recovery was smooth, with a significant reduction in hospital stay compared to traditional methods. This case highlights the potential benefits of microgranular skin grafting in managing chronic pressure injuries, offering a less invasive and more effective alternative.

## Introduction

Pressure injury (PI) represents a significant global healthcare challenge, with community-acquired cases demonstrating point prevalence rates of 0.02%-10.8% and period prevalence reaching 2.7%-86.4% [1]. Good nursing care helps prevent the occurrence of PI, slow their progression, and promote healing [2]. In fact, based on hospital and facility prevention measures, the incidence of PI in hospitalized patients is extremely low [3]. However, patients with spinal cord injuries/diseases who are restricted in their ability to move independently and are bedridden at home are highly susceptible to PI, often requiring hospitalization for non-healing ulcers [4,5].

When attempting to cure PI, the principles of wound management, including debridement, wound bed preparation, and new technologies involving the alternation of wound physiology to promote healing, are crucial [6,7]. Stages I and II PI are generally treated conservatively, while stages III and IV are usually recommended for surgical treatment [8]. Microgranular skin grafting is a surgical treatment option suitable for chronic wounds and is a technique with advantages [9]. Microskin grafting has been applied in China for over 30 years, yet the use of skin from wound edges for a type of microskin grafting procedure is limited, particularly in the objective wound healing scoring for chronic PI. This case report details microskin grafting application in a chronic stage III sacral PI unresponsive to conventional wound management in a spinal cord injury patient. It demonstrates the efficacy and advantages of this technique for treating stage III sacral PI.

## Case presentation

Patient information

We report the case of a 49-year-old male who was admitted on November 21, 2024, with a history of sacral PI for over a year, which had worsened over the past two months.

Clinical findings

At the age of 34, the patient suffered a severe car accident, resulting in injuries to the thoracic (T12) and lumbar (L1-L2) vertebrae. He underwent pedicle screw fixation surgery at a local hospital and has been bedridden since the procedure. Due to improper home care, the patient developed redness, ulceration, and exudation in the sacral area. He contacted his family doctor for PI management measures and implemented conventional management with disinfection and bandaging. However, the wound symptoms did not improve, and the wound gradually enlarged and further necrosed into deeper tissues.

Diagnostic assessment

On physical examination, the vital signs were as follows: temperature, 36.8°C; blood pressure, 114/77 mmHg; heart rate, 104 beats per minute; and respiratory rate, 20 breaths per minute. The patient exhibited absence of abdominal wall reflexes and bilateral patellar tendon reflexes, accompanied by significant muscle atrophy in the lower extremities. Neuromuscular assessment revealed complete paralysis of both lower limbs, graded as ‌0/5 on the British Medical Research Council (MRC) scale‌ (no detectable muscle contraction palpated or observed during maximal voluntary effort. This information is licensed under the Open Government Licence v3.0. https://www.nationalarchives.gov.uk/doc/open-government-licence/version/3/) [10]. A 4.0×3.0 cm chronic ulcer was observed in the sacral area, with partial necrosis of the granulation tissue, red and white intermixed appearance, severe contamination, significant exudation, purulent discharge, tissue edema, and inflammatory infiltration. Using the Pressure Ulcer Scale for Healing (PUSH) (This information is licensed under the NPUAP guidelines. https://npiap.com/page/PUSHTool), which scores from 0 to 17 with higher scores indicating greater ulcer severity [11], the patient scored 12 points. No subcutaneous sinus tracts were observed. Hematological (Table 1) and biochemical (Table 2) showed no abnormal results. Fasting blood glucose was 5.20 mmol/L (Table 2), and wound tissue culture revealed *Proteus mirabilis* (+) (Table 3).

**Table 1:** Hematological parameter values.

Categories	Abbreviations	Test value	Reference values	Units
White blood cell count	WBC	5.52	4.00-10.00	10^9^/L
Neutrophil percentage	Neu%	66.8	50.0-70.0	%
Lymphocyte percentage	Lym%	25.4	20.0-40.0	%
Monocyte percentage	Mon%	5.3	3.0-12.0	%
Eosinophil percentage	Eos%	2.3	0.5-5.0	%
Basophil percentage	Bas%	0.2	0.0-1.0	%
Neutrophil count	Neu#	3.69	2.00-7.00	10^9^/L
Lymphocyte count	Lym#	1.41	0.80-4.00	10^9^/L
Monocyte count	Mon#	0.28	0.12-1.20	10^9^/L
Eosinophil count	Eos#	0.13	0.02-0.50	10^9^/L
Basophil count	Bas#	0.01	0.00-0.10	10^9^/L
Red blood cell count	RBC	4.60	4.00-5.50	10^12^/L
Hemoglobin	HGB	121	120-160	g/L
Hematocrit	HCT	38.4	40.0-54.0	%
Mean corpuscular volume	MCV	83.3	80.0-100.0	fL
Mean corpuscular hemoglobin	MCH	27.9	27.0-34.0	pg
Mean corpuscular hemoglobin concentration	MCHC	321	320-360	g/L
Red cell distribution width-coefficient of variation	RDW-CV	13.8	11.0-16.0	%
Red cell distribution width-standard deviation	RDW-SD	48.3	35.0-56.0	fL
Platelet count	PLT	219	100-300	10^9^/L
Mean platelet volume	MPV	9.4	6.5-12.0	fL
Platelet distribution width	PDW	16.1	9.0-17.0	
Plateletcrit	PCT	0.205	0.108-0.282	%

**Table 2:** Biochemical parameter values.

Categories	Abbreviations	Test value	Reference values	Units
Alanine aminotransferase	ALT	14	0-40	U/L
Aspartate aminotransferase	AST	15	0-50	U/L
Alkaline phosphatase	ALP	108	40-150	U/L
Total protein	TP	67.5	60.0-85.0	g/L
Albumin	ALB	38.8	35.0-55.0	mg/L
Globulin	GLB	28.6	20.0-40.0	mg/L
Albumin/globulin ratio	A/G	1.4	1.0-2.5	
Potassium	K	3.52	3.50-5.50	mmol/L
Sodium	Na	139.7	135.0-145.0	mmol/L
Chloride	Cl	105.6	96.0-108.0	mmol/L
C-reactive protein	CRP	8.5	0.0-10.0	mg/L
Fasting blood glucose	FBG	5.20	3.89-6.11	mmol/L

**Table 3:** Bacterial identification results. Laboratory findings confirm *Proteus mirabilis* infection. Cefuroxime sodium demonstrates susceptibility against this pathogen. MIC: minimal inhibitory concentration.

Categories	Test value	Reference values	Units
Proteus mirabilis	+	Positive ornithine decarboxylase activity confirmed by purple color change	
Antimicrobial agent (cefuroxime sodium salt )	Sensitive	MIC ≤ 4	µg/mL

**Figure 1:**
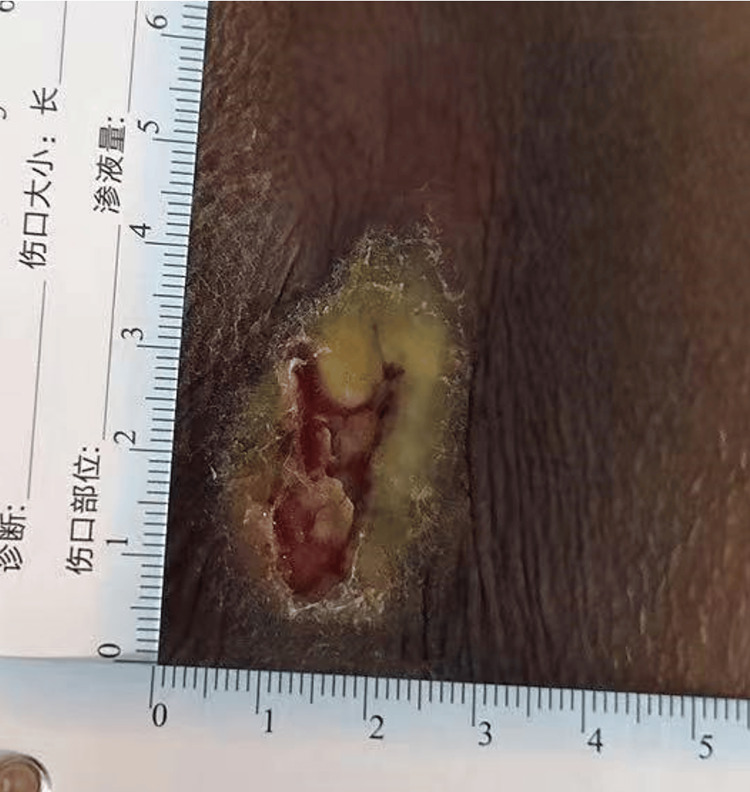
Sacral PI with erythematous ulceration and exudate (day 1). PI: pressure injury.

Sacral III stage PI with infection was diagnosed based on clinical symptoms and confirmed after debridement (Figure 1).

Therapeutic intervention

**Figure 2:**
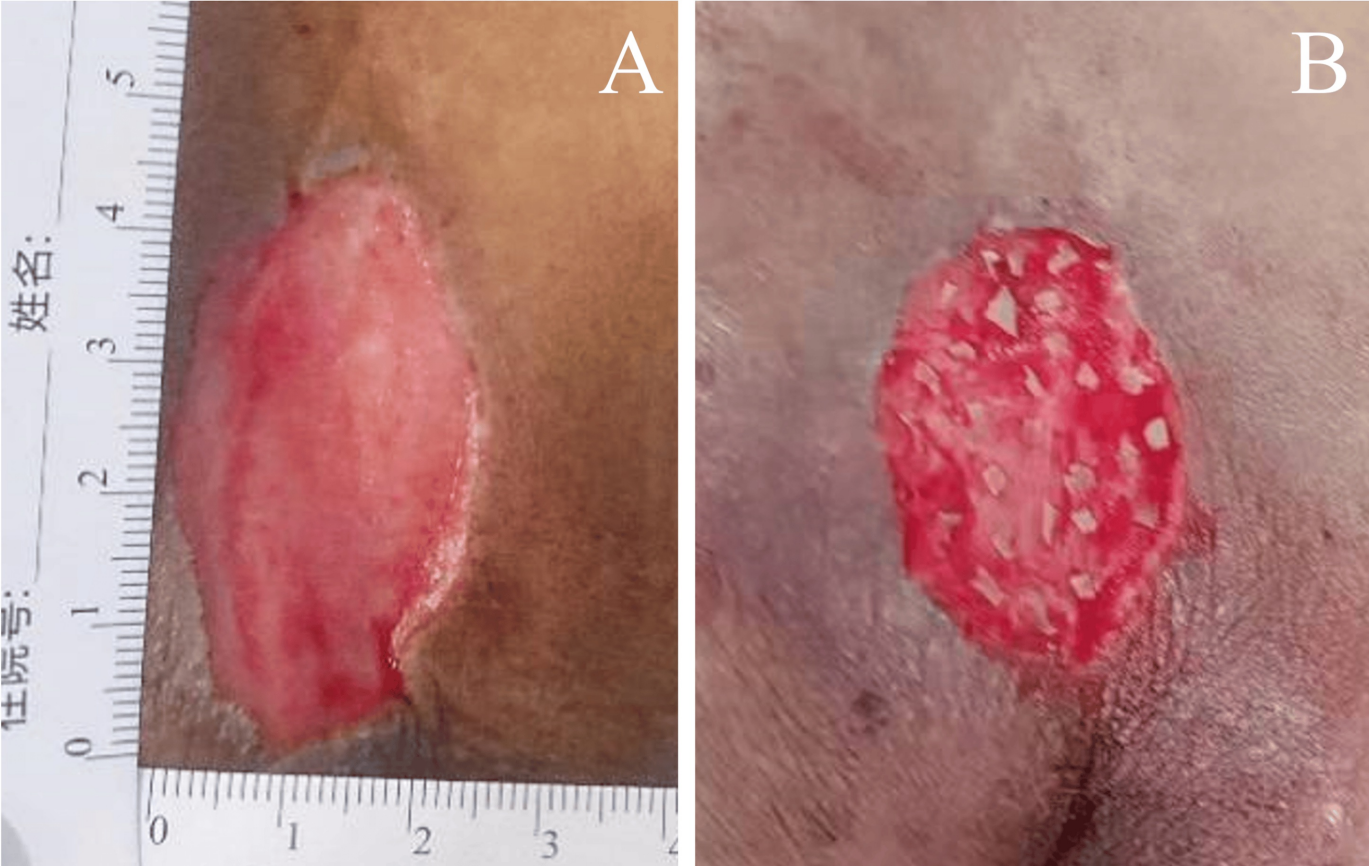
Application of autologous micrografts for wound closure‌. A, Prior to microskin grafting, complete removal of necrotic tissue and exudate from the wound bed was achieved (day 13). B, Postoperative status of microskin grafting to the wound (day 15).

On the second day of admission, debridement was performed on the PI site. The patient received intravenous cefuroxime sodium 1.5 g every 12 hours as targeted therapy for a documented *Proteus mirabilis* infection (Table 3). On the day 8 of dressing change, the wound showed a small amount of necrotic tissue and aged granulation. Ultrasound debridement was performed twice. Following completion of preoperative preparations, no necrotic tissue was present, exudate had diminished, and fresh granulation tissue was evident on the wound surface (Figure 2A). On day 18, an appropriate amount of full-thickness skin was harvested from the periphery of the patient's wound edge. The harvested skin was cleansed to remove adherent fat and other debris. After cleaning, the tissue was minced into (1.0-2.0) mm x (1.0-2.0) mm particles as uniformly as possible using scissors and then immersed in 0.9% sodium chloride solution. After confirming hemostasis within the wound, the minced skin grafts were distributed evenly across the wound surface, spaced approximately 5 mm apart (Figure 2B).

The rationale for prioritizing granular skin grafting over flap reconstruction in this case is to minimize risk and optimize medical resource utilization by avoiding unnecessarily complex surgical interventions while ensuring effective repair.

Postoperative care

**Figure 3:**
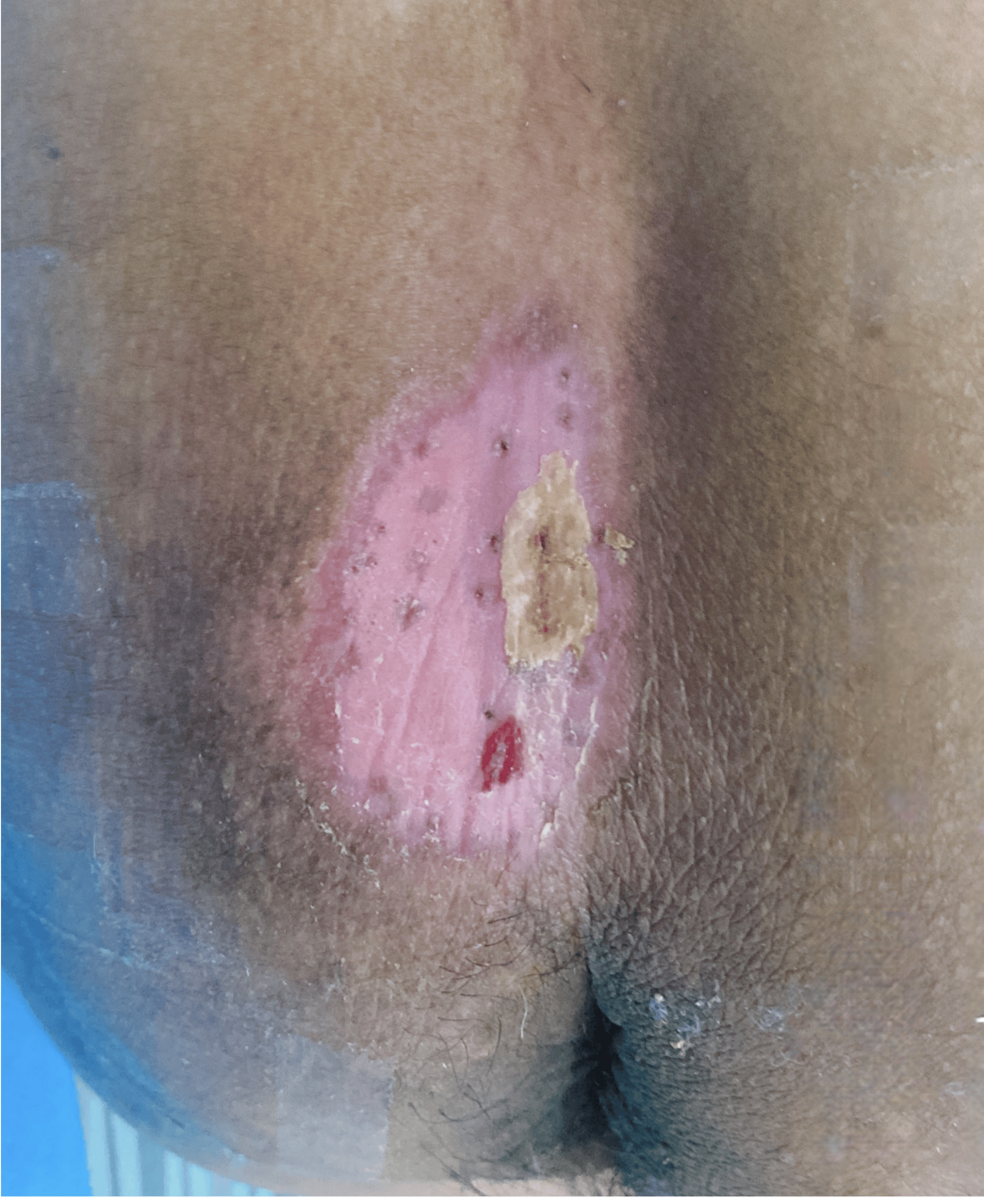
Wound healing with complete epithelialization (day 27).

The patient was advised to follow a high-protein (10.5-12.5 MJ), high-fiber diet; avoid prolonged pressure on the affected area by turning every two hours with a turning schedule card; use antibedsore air mattress Cofoe® (Cofoe Medical Technology Co., Ltd., Changsha, China) to reduce pressure; maintain skin moisture balance with moist burn ointment Meibao® (Shantou MEBO Pharmaceutical Co., Ltd., Shantou, China); and regularly check the wound healing until complete epithelialization was achieved, and the patient was restored to a sitting position (Figure 3).

Outcome

**Figure 4:**
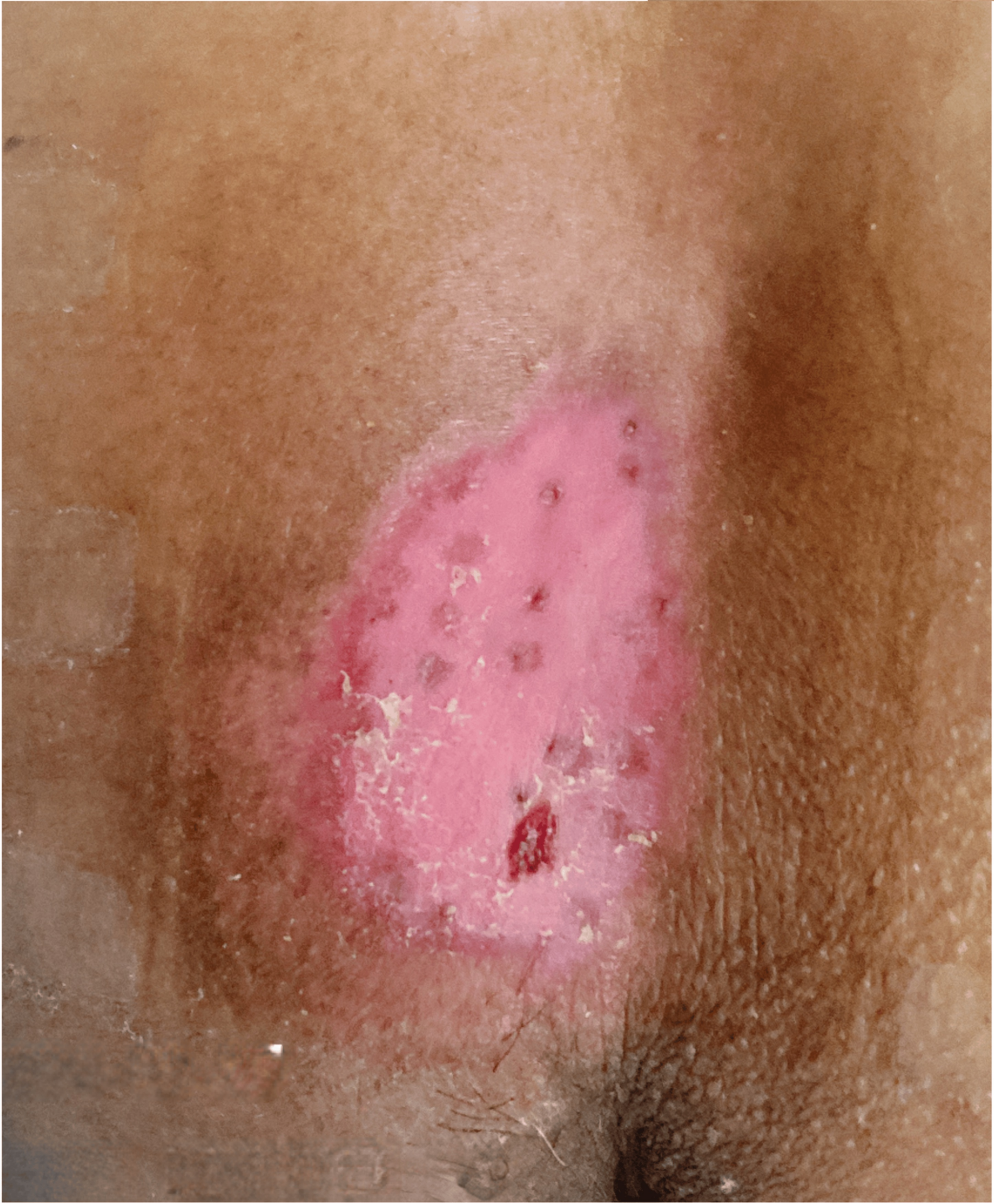
Achieved primary endpoint: medically cleared for discharge (day 30).

Microparticle skin transplantation demonstrates clinical efficacy, with complete ulcer epithelialization achieved within 30 days (Figure 4); the patient scored 0 on the PUSH.

At the six-month postoperative follow-up, the patient remained free from recurrent pressure injury in the sacrococcygeal region. The scar was evaluated using the Scar Cosmesis Assessment and Rating (SCAR) Scale (This information is licensed from Mapi Research Trust (via ePROVIDE), no. 251363), where scores range from 0 (best possible scar) to 15 (worst possible scar) [12]. The final assessment score was 5.

## Discussion

PIs are localized skin injuries caused by moisture, friction, shear, or prolonged pressure, particularly in bony prominences such as the sacrum, coccyx, and heels [13]. Although PIs are considered preventable, patients with limited mobility, such as those with spinal cord injuries and those undergoing prolonged surgery, are particularly susceptible to PIs during static positioning [14,15]. The prevalence of PI in paralyzed patients is high, with 33.9% in quadriplegics, 47.4% in paraplegics, and 9.6% in hemiplegics [14]. Additionally, approximately 6% of spinal cord injury patients experience recurrent PI [15].

The pathogenesis of PI primarily involves ischemia-reperfusion injury, impaired lymphatic drainage, cell deformation, excessive apoptosis, and disruption of the extracellular matrix (ECM), leading to chronic inflammatory states and impaired healing [16,17]. Given the unique factors in the mechanism of PI, more specific preventive and therapeutic interventions are required.

PI staging is defined based on the severity of skin, tissue, and muscle damage. Specifically, once PI reaches stage III, extending through the skin into deeper tissues and fat but not reaching muscle, tendon, or bone, the treatment process becomes prolonged. Previous studies have indicated that stage II PI wounds take an average of one month to heal, while stages III or IV PI wounds may take up to four months [18].

Conservative treatment is ineffective for stage III or IV PI, and extensive debridement and adequate flap reconstruction become inevitable [8]. On the other hand, there is still controversy regarding the key factors, namely flap selection and postoperative care (short-term hospitalization in a surgical ward or long-term stay in a rehabilitation ward) [15].

The goal of surgical reconstruction is to effectively cover the wound with vital tissue. We must fully consider the risk of recurrence in spinal cord injury patients and preserve the donor site for further simple reconstruction options [15]. Due to its high skin expansion rate, microgranular skin grafting is widely used, which can maximize the use of limited skin sources to close larger wounds [9]. In this case, we chose microparticle skin transplantation from the wound edge and observed that the skin island formed by microparticle skin accelerated wound healing, the recovery was relatively stable, and the hospital stay was shortened by one month. This surgical method has the advantages of reduced trauma, low surgical requirements, and stable therapeutic effects.

Regarding postoperative care, we advocate that patients with spinal cord injuries and PI should be hospitalized until the wound is completely healed and the patient is restored to a sitting position to prevent immediate complications and the tendency for recurrence. As is well known, spinal cord injuries can lead to severe sequelae and significant economic burdens on society [19]. In China, this type of care can be fully covered by medical insurance funds, and patients and their families will benefit from reduced economic expenditures.

## Conclusions

This study evaluated the application of wound edge-derived microskin grafts in the treatment of stage III sacral PI in paraplegic patients. The results demonstrated that the grafts accelerated healing, thereby eliminating the need for harvesting skin from different areas. This technique is a minimally invasive and efficient method suitable for the repair of PI. Comprehensive postoperative care ensured recovery and minimized the risk of recurrence. It is evident that this treatment is highly effective for such wounds, alleviating patient suffering while also saving time and reducing economic costs. Currently, this study includes only one case; future research will expand the number of cases and conduct controlled studies.
